# Expectations for a co-designed virtual immersive program for older adults with dementia in long-term care: a qualitative study with residents, family, and staff

**DOI:** 10.3389/frdem.2026.1735564

**Published:** 2026-02-12

**Authors:** Mary Van, Joey Oi Yee Wong, Albin Soni, Bonnie Huynh, Shambhavi Arora, Jim Mann, Christine Wallsworth, Lily Wong, W. Ben Mortenson, Angelica Lim, Lillian Hung

**Affiliations:** 1 Faculty of Science, University of British Columbia, Vancouver, BC, Canada; 2 School of Nursing, University of British Columbia, Vancouver, BC, Canada; 3 Faculty of Biomedical Engineering, University of British Columbia, Vancouver, BC, Canada; 4 Faculty of Arts, University of British Columbia, Vancouver, BC, Canada; 5 Department of Occupational Science and Occupation Therapy, University of British Columbia, Vancouver, BC, Canada; 6 School of Computing Science, Simon Fraser University, Burnaby, BC, Canada

**Keywords:** co-creation, collaborative research, design thinking, long-term care, neurodegenerative disease, patient and public involvement, technology

## Abstract

**Introduction:**

Virtual immersive programs (VIP) have been found as a recreational medium with positive impacts on the quality of life for older adults living with dementia in long-term care (LTC). While studies have explored the adoption of a VIP in LTC and suggested the need for tailored programs, there is a paucity of studies that have investigated the expectations of end-users of VIP in LTC. As part of a larger study to co-develop a VIP in LTC settings guided by the design thinking framework, this paper reports the purpose of our study, focused on the first two stages of that framework (i.e., empathy and define), which was to explore expectations prior to implementation of VIP from the perspectives of residents, family members, and staff in Canadian LTC home settings.

**Methods:**

We conducted qualitative interviews and focus groups at one LTC home with 14 residents, six family members, and six staff members. We used reflexive thematic analysis to identify key expectations regarding content and delivery, positive outcomes, and concerns of a VIP in LTC.

**Results:**

We identified five themes (1) Personalized and engaging content, (2) A world without boundaries, (3) Shared experiences and care support, (4) Challenges of transitioning back to actual reality, and (5) Technology and usability barriers.

**Conclusion:**

Our findings offer useful insights and practical tips through the recommendations IMAGINE in co-designing technology that is appropriately tailored to residents, staff, and family members in LTC. This study provides foundational knowledge for the co-design of VIPs by highlighting end-user expectations prior to implementation, an often-overlooked stage in technology development.

## Introduction

Dementia is one of the most common progressive neurodegenerative diseases characterized by cognitive decline that interferes with independent, social, or occupational functioning (Gale et al., 2018; Cao et al., 2020). As of 2020, an estimated 47 million people live with dementia worldwide (Arvanitakis et al., 2019) and this number is expected to increase to 131 million by 2050 as the population ages (Arvanitakis et al., 2019), with various factors including age, gender, and geography influencing disease prevalence (Cao et al., 2020). With the nature of the disease progressively declining mental capacity, people living with dementia often transition to living in long-term care (LTC) for their complex care needs (Sm-Rahman et al., 2022). About 42% of older adults with dementia ages 80 and above, and one-third of older adults under 80 with dementia live in LTC (Canadian Institute for Health Information, 2025).

Beyond providing basic care, LTC homes are encouraged to explore various innovations that maximize the independence, autonomy, personal fulfilment and human dignity of individuals (World Health Organization, 2021; Zhang et al., 2023). Contrasting from television, Virtual Immersive Programs (VIP) offer a three-dimensional, virtual environment that users can interact with in two forms: a headset with fully immersive visual and auditory features, or via multiple surrounding screens or projections (Hamad and Jia, 2022). VIPs have been suggested to actively engage participants more effectively than television because of its ability to allow first-person perspective observation while incorporating social cues and spatially adapted multisensory experiences that create a physical sense of presence within the scenario (Dang et al., 2018; Lindner, 2021; Yoo and Ohu, 2024). Although designed for video gaming initially, VIPs have diverse applications in healthcare settings including education, training, simulations, and facilitating exercises (Hamad and Jia, 2022). In addition to VIPs providing an opportunity to reduce loneliness and social isolation for older adults in LTC (Hung et al., 2023b), it can also promote overall mental wellbeing and social engagement, which is often poor in LTC homes (Kwan et al., 2023; Lin et al., 2018). One suggested advantage of a VIP is the ability to bypass barriers caused by physical disabilities and environmental restraints and allows older adults to experience therapeutic elements, including reminiscence (Hsieh and Wang, 2003), nature-based, and outdoor activities (Kwan et al., 2023). Qualitative studies have additionally suggested that VIPs promote residents’ sense of self through reminiscence and storytelling (Wong et al., 2025), as well as strengthen connections between staff and residents (Hung et al., 2023a). However, concerns have been raised about practical barriers including equipment cost, patient suitability, the need for ongoing staff training, staff facilitation, and the acceptance of and attitudes towards technology in healthcare settings, which can make the application of such programs challenging (Hung et al., 2023a; Aderinto et al., 2023).

LTC residents who have experienced forms of a VIP have expressed a desire for tailored videos. Varied content displaying contextually and culturally relevant symbolism are important factors in creating a positive experience (Hung et al., 2023a). In a quantitative study examining the acceptance of VIP in LTC, it was found that the successful integration of VIP depends on a holistic understanding of patient needs and tailoring VIP designs to accommodate for preferences and potential participation challenges (Shin et al., 2023). The value of having personalized VIPs catered to personal preferences has the potential to elicit discussion of past experiences, emotions, and has been shown to improve the quality of life and mental wellbeing of older adults (Hosseini et al., 2024), including those with dementia (Park et al., 2019), and those living in LTC (Syed Elias et al., 2015). Moreover, a study on older adults residing in LTC found that the use of VIP in the delivery of reminiscence therapy was greatly effective in treating symptoms of apathy (Saredakis et al., 2020). Altogether, these findings suggest a need for tailored, patient-centred programs in making VIP an effective means of improving residents’ quality of life in LTC settings.

Previous literature emphasizes co-design as a participatory approach that actively involves residents, family members, and staff in shaping healthcare technologies (Guo, 2023). This engagement has been shown to improve functionality and adoption of technologies by aligning solutions with real-world needs and practices (Howard et al., 2025). One study recommended that co-design fosters meaningful engagement, enhances user satisfaction, and contributes to positive outcomes such as improved communication, motivation, and a sense of accomplishment (Boamah et al., 2025). For older adults and those with dementia, co-design tailors interventions to individual preferences, addressing the persistent issue of low technology uptake (Langer and Merkel, 2023). Despite these benefits, several barriers in healthcare limit effective co-design. A key challenge is the lack of a standardized definition and methodology for co-design, leading to inconsistent implementation (Langer and Merkel, 2023). Organizational barriers such as staff workload, time constraints, and limited technical skills; further reduce engagement (Ramos et al., 2020). In LTC, staff often experience stress and burnout, making sustained participation difficult (Ramos et al., 2020). Older adults with dementia face unique difficulties in articulating preferences, which requires researchers to adapt methods carefully. Additionally, scaling co-design approaches remains challenging, particularly when regulatory complexity, lack of shared language, and technical failures in digital platforms are considered (Howard et al., 2025).

While co-design has been applied to mobile apps and assistive devices, there is limited research on how residents, staff, and family members are jointly engaged in designing tailored VIPs in LTC. Most evaluations emphasize usability and acceptance rather than long-term health and well-being outcomes, leaving a gap in evidence for the broader impacts of co-designed VIP interventions. Although VIP interventions in LTC have demonstrated benefits such as reducing loneliness and enhancing engagement, the majority of research remains outcome-focused, evaluating changes after programs are introduced. This ignores a major gap stemming from lack of co-design approaches: little is known about what residents, family members, and staff anticipate from VIPs prior to their design and implementation. Our study addresses this gap with the research question: What are the expectations from residents, family members, and staff for a VIP for older adults with dementia in long-term care?

## Methods

### Design thinking methodology

**Figure 1:**
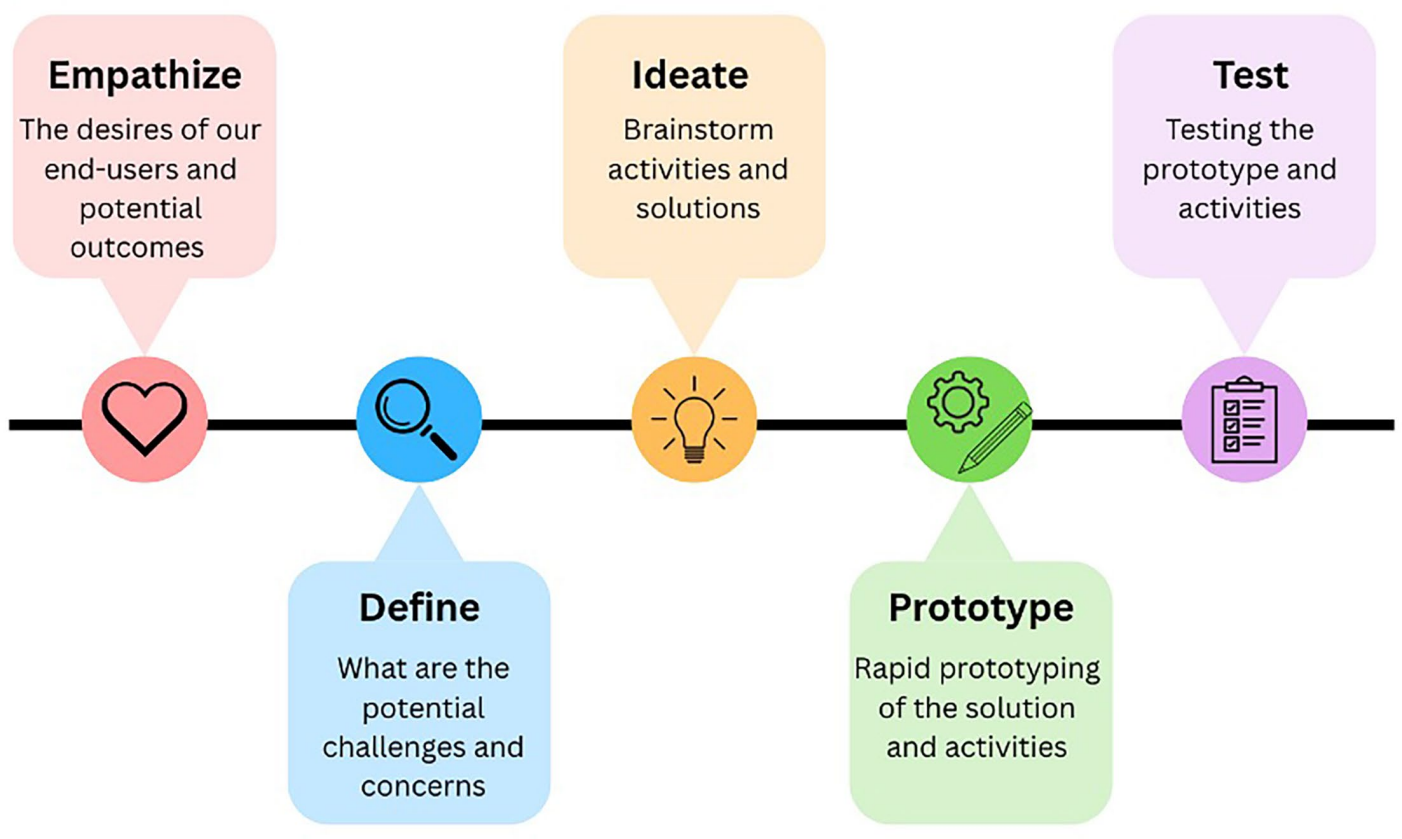
Design thinking methodology.

Our study was guided by the design thinking methodology to ensure the co-created VIP in LTC prioritizes the needs, desires and challenges of the end-users (i.e., residents, family members, and staff) (Roberts et al., 2016). Design thinking methodology offers a systematic and collaborative approach to foster innovative and team-generated solutions that guides our research team to effectively engage residents, family members, and staff from the beginning of the study (Lorusso et al., 2021; Pepping et al., 2023). All participants are part of the co-design team. The design thinking process is constantly evolving and has a variety of models (Lorusso et al., 2021). Our study followed the design thinking process proposed by Stanford University’s Hasso Plattner Institute of Design (2010). The five stages include (1) empathize (the desires of our end-users and potential outcomes), (2) define (what are the potential challenges and concerns), (3) ideate (brainstorm solutions and activities in the virtual immersive programs), (4) prototype (rapid prototyping of the solution and potential activities), and (5) test (test the prototype and activities) (see Figure 1). The design process is iterative. This paper will focus on the first and second stages—empathize and define.

### Sampling and recruitment

Participants were recruited by convenience sampling (Stratton, 2021). For resident participants, they were recruited based on the inclusion criteria: (1) residents living in the long-term care home of the study and (2) the ability to speak and express themselves. Family caregiver participants were recruited if (1) they are the family member of a resident staying at the long-term care home of the study and (2) they can speak, read and understand English. For staff participants, they were recruited if they were (1) regular or casual frontline staff working at the long-term care home of our study, (2) working in a discipline that provides direct care to residents or manages residents’ care (e.g., nurses, recreation staff, decision makers, or care aides) and (3) able to speak, read and understand English. The participants were excluded if they have (1) severe visual or auditory impairment, or (2) are sensitive to motion sickness and have a history of seizures or epilepsy. The frontline staff members (e.g., the recreation staff member and director of care) helped identify potential participants, including residents, family members and staff members at the long-term care site. The principal investigator, LH, followed up with the family member with phone calls to introduce them to the research and confirm recruitment. The research team project coordinator and trainee, JW, followed up with the residents and staff members in person to introduce them to the research and confirm recruitment.

### Setting

Data collection was conducted in one LTC home in an urban area of Metro Vancouver, British Columbia, Canada. The care home is a not-for-profit care home with 230 beds, with the average age of residents being 85 years old. Sixty percent of the residents are living with mild to severe dementia, and about half of the residents are in wheelchairs.

### Data collection

For the empathize and define stages, two focus groups and six individual interviews were conducted from November 2023 to January 2024. After reflecting on the group dynamics in the first focus group, our team decided to conduct separate interviews with staff to ensure opportunities for all partners to share their insights, as well as to accommodate their work schedules. The first focus group was done in November 2023 and involved eight residents, six family members and three staff members. The second focus group involved participants who rejoined from the first focus group, which consisted of six residents and four family members. The second focus group along with six staff interviews were done in January 2024. Each focus group lasted for 60 min. Two residents and family members did not attend the second focus group because they were unwell or had other scheduling conflicts.

**Figure 2:**
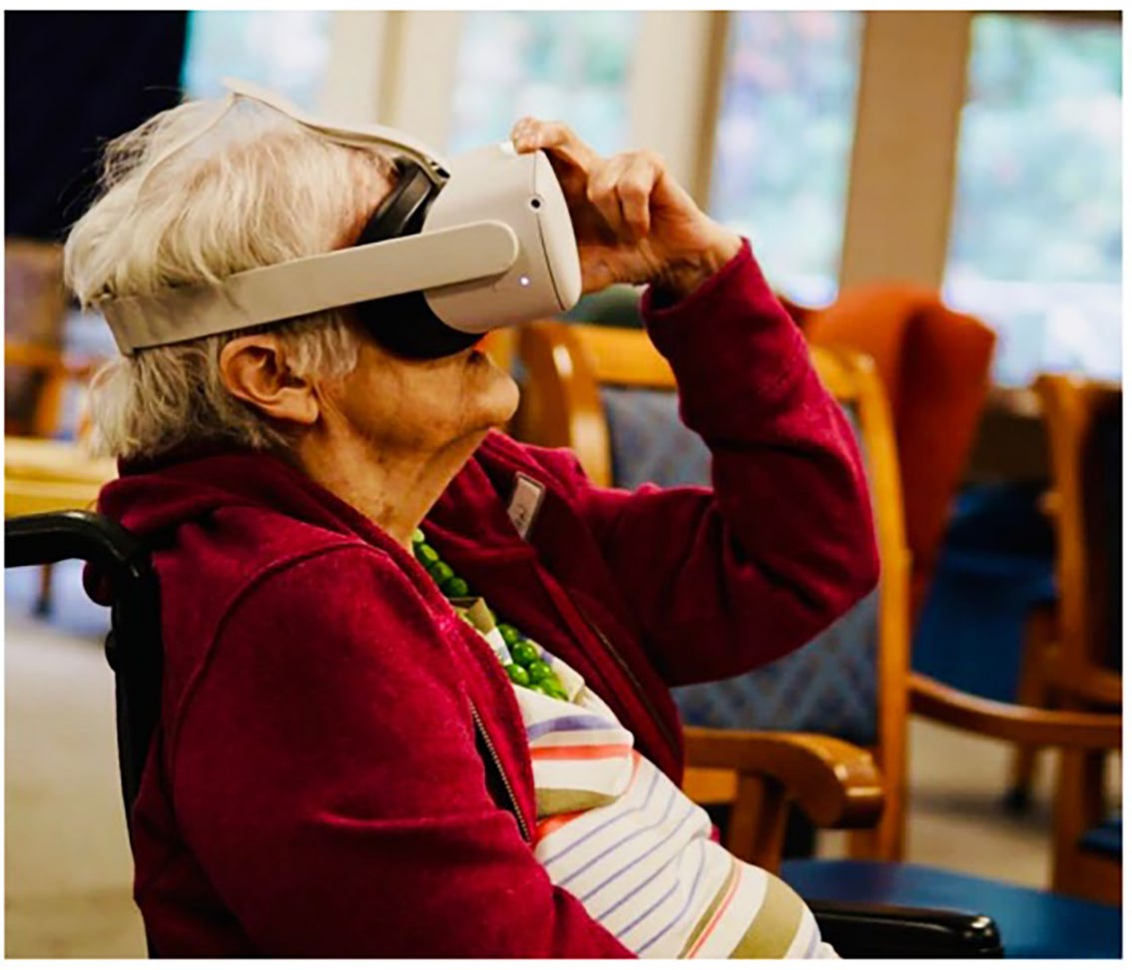
A resident using an Oculus 2 headset at the beginning of the focus group.

At the beginning of the interviews and focus groups, JW explained the project and what VIP was. We define VIP as fully immersive headsets and semi-immersive projections to accommodate for populations in LTC who may experience discomfort with headsets. After explaining what VIP was, participants would have the opportunity to try the virtual reality headsets and watch a one-minute video of their choice among three videos prepared by our research team, including a visit to a local food market in Vancouver, a visit to a Chinese restaurant and a trail walk in Vancouver. The headsets used were Oculus 2 headsets (see Figure 2). In the interviews and focus groups, we followed a semi-structured interview guide with the following questions:

1. What do you imagine the virtual immersive program may look like for people with dementia in long-term care homes? (Focus group 1)
2. What positive experiences or outcomes do you expect? (Focus group 1)
3. What concerns do you have in terms of having a virtual immersive program for people with dementia in long-term care homes? (Focus group 2)

All interview and focus group audio recordings were transcribed verbatim.

### Ethical consideration

Ethical approval for this study was obtained from the University of British Columbia Research Ethics Board (Ethics ID: H23-01481). We implemented a process for ongoing consent and assent as per best practice (Diaz et al., 2025). All participants provided written informed consent. We obtained consent from the resident’s substitute decision-maker and assent from the resident themselves. Crucially, we treated this initial consent as a starting point. We continuously monitored for verbal and non-verbal signs of assent (e.g., nodding, smiling, active participation) and dissent (e.g., verbal refusal, turning away, pushing the headset away, expressing discomfort) throughout the data collection session. Participants were explicitly informed that they could pause or withdraw at any time without any consequence to their care. Participants also provided verbal consent for audio recordings and photo taking. If the participant showed disinterest or declined participation, their decision was honored without any attempt to persuade or coerce them into the interviews of focus groups. Fieldnotes were taken if the interview was consented to, but audiotaping was declined. Verbal consent was obtained for the pictures used in the paper. All identifying information in the pictures included in the paper was blurred. Pseudonyms are used in this paper to protect the anonymity of the participants and the care homes.

### Data analysis

We conducted reflexive thematic analysis and followed the six phases to analyze the data collected from the interviews and focus groups (Braun and Clarke, 2021) to identify expectations, positive outcomes, and concerns of a VIP in LTC. (1) Two research assistants (MV and AS) familiarized themselves with the transcripts by reading and re-reading them. (2) MV and AS identified meaningful data segments and generated initial codes. (3) MV and AS shared initial codes and generated initial themes. (4) The research team, including the research assistants, the supervising researcher, LH and the other team members, reviewed and discussed the preliminary themes. (5) The research team refined and finalized our themes. (6) MV and AS drafted the findings. See Table 1 for examples of coding.

**Table 1:** Examples of coding.

Themes	Codes	Quotes
Opportunities for communal experiences and support for care	Multiple residents engaging together Preference for projector	I think [the projector is] a good idea, maybe also since it’s there if you gather all the residents with the same experience or same background, and maybe that can also be positive as something that [everyone] can relate to, and they can talk about it more.
Ability to bring and connect residents to a world without boundaries	Remembering locations outside facility in community Desire for new experiences	I feel like to see a lot of the world because I’ll never see it…Get to see some places like me for never seen them…Like that Chinese restaurant. I’ve never seen it.

### Research team

The research was led by Principal Investigator LH, an experienced researcher in the field of aging and technology, who provided overall supervision and methodological guidance. We included members with diverse expertise in our research team. There were patient and family partners with lived experience of dementia, and researchers and trainees from diverse disciplines of nursing, science, arts, biomedical engineering, occupational therapy, and computer science.

### Rigour

To enhance the credibility of our study, we engaged one person living with dementia not residing in LTC and two family partners with lived experiences at every stage of the research. They were involved in interview question development during monthly research meetings on Zoom, data collection sessions, and data analysis meetings over a period of 3 months on Zoom to ensure our work was relevant to the topic and ethical. Second, as a research team, the research assistants involved in the data collection sessions wrote research journals after each interview and focus group to reflect on the data collection process, practising reflexivity on how the sessions were carried out, the challenges and our individual assumptions stemming from our diverse social backgrounds. During the team meetings, the research team would discuss and challenge each other’s assumptions. To enhance the rigour and clarity of our qualitative reporting, we used the COREQ checklist to guide reporting of our study procedures and findings. See Appendix I for the COREQ checklist.

## Results

Twenty-six participants, including 14 residents, six family members, and six frontline staff, participated in our study. See Table 2 for the demographic characteristics of the participants. Over half of participants were residents of Caucasian ethnicity who were at least 71 years old; the majority of residents were over the age of 80 and utilized walking assistance devices. Residents and family caregivers were predominantly female. Staff ranged between 41 and 70 years old and were from diverse disciplinary backgrounds.

**Table 2:** Demographic information of participants (*N* = 26).

Residents		Family caregivers		Staff	
Gender					
Male	5 (35.7%)	Female	6 (100%)	Male	3 (50%)
Female	9 (64.3%)			Female	3 (50%)
Age group					
71–80 years	4 (28.6%)	31–40 years	2 (33.3%)	41–50 years	2 (33.3%)
80 + years	10 (71.4%)	41–50 years	1 (16.7%)	51–60 years	3 (50%)
		61–70 years	1 (16.7%)	61–70 years	1 (16.7%)
		71–80 years	2 (33.3%)		
Ethnicity					
White	13 (92.9%)	White	3 (50%)	White	4 (66.7%)
Middle Eastern	1 (7.1%)	Middle Eastern	1 (16.7%)	Southeast Asian	1 (16.7%)
		Southeast Asian	2 (33.3%)	East Asian	1 (16.7%)
Walking assistance				Staff roles	
In wheelchairs	11 (78.6%)			Pastoral care staff	1 (16.7%)
No walking assistive devices	3 (21.4%)			Care aid	1 (16.7%)
				Leadership administrator	1 (16.7%)
				Recreation Assistant	1 (16.7%)
				Recreational Therapist	1 (16.7%)
				Nurse	1 (16.7%)
Total	14 (53.8%)		6 (23.1%)		6 (23.1%)

The reflexive thematic data analysis generated the following five themes: (1) Personalized and engaging content, (2) A world without boundaries, (3) Shared experiences and care support, (4) Challenges of transitioning back to actual reality, and (5) Technology and usability barriers. Table 3 summarizes the themes and which themes were commonly expressed among participants.

**Table 3:** Themes commonly expressed by respective participant groups.

Theme	Staff	Family	Residents
Personalized and engaging content	X	X	X
A world without boundaries	X	X	X
Shared experience and support for care	X	X	X
Challenges transitioning back to actual reality	X	X	
Technology and usability barriers	X		

### Personalized and engaging content

Appreciating the potential of VIPs to recreate specific experiences, residents had expressed the desire to be immersed in activities that they enjoyed, as well as given the opportunity to partake in these activities that they may no longer be able to do as a result of physical or cognitive limitations, or residency in LTC. The inclusion of familiar faces in the videos was also an essential factor in making the VIP experience more interactive and engaging.

A key expectation of an appropriate VIP is to ensure that content is tailored to residents’ unique preferences, experiences, and backgrounds as stated by one staff member, “I think [a VIP should] be individualized to the residents with their past and history.” For example, Ivan, a resident who had previously played hockey and enjoyed watching hockey games shared that being able to see a program on an enlarged screen, where it seemed that they were a player in the hockey game in real time, excited him:

When I played hockey and [it] expected me to basically pursue the puck, I would get it and pass it or score a goal or do something. Yea, absolutely I would want to [experience it again]… I would want to be part of the games to go from point A to point B, like here and there, and my focus would be to put the puck in the net.

For residents who previously engaged in sports and continue to especially enjoy sports, VIP can provide residents the experience of being players in sports games.

Other residents also communicated that they wanted content that enabled artistic appreciation by showcasing performances with music and dancing. A resident, Rosie, expressed, *“I like dancing*… *[I want to see] tap dancing, ballet dancing, all kinds of dancing*… *a lot of figure dancing would interest me.”* Some residents stated that cheerful music and dancing from an era relevant to them could actively engage and encourage other residents to dance along when seeing individuals in the VIP dancing. There were residents who preferred to see videos of day-to-day activities that they used to frequently participate in. For example, Robert shared how he enjoyed driving because it was an important aspect of his life. He thus expressed that he would like to drive again using the VIP:

Facilitator: Do you want us to film the car or inside the car?

Robert: Inside the car.

Facilitator: Do you want to be the driver?

Robert: (nodded) And nobody is telling me what to do!

This example further demonstrates how VIPs that are well-tailored according to the preferences of residents could potentially provide a sense of autonomy as well as foster personal meaning and engagement for residents in LTC settings.

Furthermore, residents shared how VIP content would be more engaging if individuals being shown in the programs were familiar and relevant to them, such as the residents themselves, other residents, or their loved ones. A resident, Paulina, shared that the videos would be boring without displaying familiar faces:

Facilitator: If there are some familiar faces that they [residents] see in the video, then what would they feel?

Paulina: Well, if [familiar faces] were in it, they were in the picture, maybe it might be more of an interest to them.

Facilitator: Do you mean that if the residents themselves or the people watching themselves in the video?

Paulina: Yes. I would think [that is] more of an interest than just watching other [unfamiliar] people.

Participants emphasized that delivery was equally important in shaping a meaningful experience. Many residents noted that while passive watching was enjoyable, the impact of VIP would be significantly enhanced if it incorporated active physical elements. One resident, Sally, explained, *“We need to be moving, not sitting.”* This suggests that VIPs could be designed to encourage movements and support residents in staying both physically active and cognitively engaged. Another resident also appreciated an alternate viewing option of having projections on a wall or larger screen, enabling VIPs to be more inclusive for residents who may struggle to use head-mounted devices. One resident, Charlie shared, *“[The video would] be great in a big square [screen]. If you could get it that way, then you can see it a little bit more.”*

### A world without boundaries

VIPs were expected by staff, family members, and residents to provide the ability to experience both novel and familiar worlds, enabling a sense of connection both to self and others. Staff believed that VIPs could allow residents to reconnect with their previous communities and neighbourhoods, providing a brief sense of ‘home’ while at the care home. One staff member, Tom expressed that the VIP could provide individuals unable to leave their rooms the opportunity to explore what is outside the care home:

A good opportunity to visually reconnect people with stuff. Makes life a little more whole. Especially if you're bedridden, right? I mean, wouldn’t that be cool, right? For somebody who's bedridden and can’t move. Go back to the old neighbourhood.

Staff suggested that residents would be able to experience a sense of self-connection when reminiscing on their past. Another staff member, Anna commented how residents can move beyond local experiences and travel to other parts of the world, *“People come from wherever [such as] Italy, and you can take them on a trip to Italy, and they do not have to leave the facility.”*

Similar to staff, residents also echoed the sentiment of exploring what is outside the care home as a positive outcome expected of the VIPs. One resident, Peter shared how he expected the VIP to be a medium in which he could see parts of the world and community that he never had the chance to see: *“I feel like to see a lot of the world because I’ll never see it… Get to see some places like me for never seen them… Like that Chinese restaurant. I’ve never seen it.*” Furthermore, staff and family members imagined VIPs as an opportunity to provide residents novel experiences, such as extreme sports and travelling to different countries, that they might not be able to try in reality due to physical age-related limitations:

So maybe something cool like riding a ski lift or gondola, riding a motorcycle, maybe a mountain bike, I don't know, something different, or maybe being up like, the top of Mount Everest or something and work around that way. (Amanda, a staff member)

One family member, Biraj, echoed travelling as a beneficial experience for residents: “*One of the things would be travel, showing different countries and different cultures would be really fascinating with a lot of color*.” This example further displays that travelling to new parts of the world allows residents to potentially feel more connected to others and themselves as they learn about different cultures, including their own.

### Shared experiences and care support

VIPs were expected to foster social connection and support overall wellbeing among residents. The program was also imagined as a practical behavioural support tool for staff within LTC by providing distraction, comfort and positive stimulation, managing agitation, and easing transitions during difficult routines.

Participants proposed that VIPs should not be designed as solitary experiences provided in headsets only, but include opportunities for dialogue, storytelling, and collective reflection to enable social connection through mediums such as an immersive projection. One staff member, George, highlighted the potential of shared reminiscence among residents with common experiences or backgrounds. George described how the residents could experience VIP together through an immersive projection:

I think [the projector is] a good idea, maybe also since it’s there if you gather all the residents with the same experience or same background, and maybe that can also be positive as something that [everyone] l can relate to, and they can talk about it more.

A family member, Kim, also mentioned the creation of a shared space through a communal approach when using the projector: “*Having a space for them [residents] to go to and have a projector on the wall is great, and usually there will be more than one person there and other residents.”* Residents also shared how they expected VIPs to support social connections with other residents beyond verbal communications, similar to how residents had expressed motivation to dance when seeing dancing figures on the VIP. A resident, Tonya expressed a preference for singing along with other people in front of the VIP rather than by herself: “*I like to sing with other people. I’m not a singer, but if everybody sings, I’m happy with it*.”

In addition to fostering interpersonal bonds between residents, a VIP with communal delivery through projection can create an atmosphere of engagement among other relationships. A staff member, Sarah stated that the VIP could allow intergenerational interaction between residents, family and friends, or community members: “*There can be an involvement [of the residents] with the younger generation. They [the younger generation] know more about VIP. This friend or younger family member who is visiting them [the residents] can explain [VIP] to our residents, and this is a way of discussing things.*” With the VIP, residents and younger individuals could have conversations together. Residents might feel connected to younger generations while enabling younger generations to understand older people through interactions. Nonetheless, staff noted that communal formats might not always be beneficial, depending on whether the residents had formed close relationships with one another. One staff member, Yoojung, reflected, “*For social interaction…although there would be some [residents that] would prefer to do it [the VIP program] on their own.”*

Besides social engagement, staff consistently described VIPs as a promising means of supporting behaviour management in LTC environments. Behavioural responses are defined as actions, words, or gestures made by an individual with dementia in responding to negatively perceived stimuli in the environment and can be presented as aggression, restlessness, and paranoia (Alzheimer Society of Canada, 2019). When working with residents with behavioural responses, VIPs were viewed as unique tools for redirection. Staff also suggested the use of VIPs prior to behavioural escalation or during unexpected episodes of agitation, demonstrating that they envisioned the programs as both a preventive and responsive tool. One staff member, Kayla explained how VIPs were expected to provide calming effects for residents who frequently experienced agitation or distress:

I really see a potential for this kind of programme in the long-term care home, especially for behaviour concerns. We have one resident who [easily gets] agitated, but when we try this kind [of VR program], it may make him calm.

Staff also suggested how VIP could support their care such as bathing, which could be distressing for some residents living with dementia. As one staff member Zach, reflected: *“Bathing is a very difficult one for residents and then you can help them [using the VIP] be in a place that wasn’t so scary.”* By immersing residents in calming or enjoyable environments, VIP was expected to reduce resistance, agitation, and fear during these care activities, ultimately making them less stressful for both residents and care staff.

### Challenges transitioning back to actual reality

One concern shared by staff and family members about having VIPs in LTC was the transition between virtual and actual reality, which entailed difficulty taking breaks from the VIP and feelings of sensory overload for residents.

Staff demonstrated concern over residents enjoying the VIP to an extent where it became disruptive in necessary daily routines and care administered by staff, such as meals and medication administration. A staff member, Adrien vocalized the concern that residents might refuse to stop watching the VIP: *“If it were to cause them [residents] to be upset [when the staff] interrupt [them using VIP] for when they need care or medications, residents may say “no, no just leave me alone,” that could be a problem.”*

Another concern expressed by staff was the potential of VIPs in causing disorientation and sensory overload for residents. Given the immersive visual and audio effects of these programs, some residents might feel overwhelmed. One staff member, Sam stated,

It [The immersive experience] could initially be a scary thing, right? Because all of a sudden, people, they’re immersed in a world where they can see [things] they couldn’t see before and there might be a bit of a sensory overload kind of issue initially. And so I would think that might be a little disconcerting.

A family member, Ella also echoed this concern that residents may experience difficulties adapting their sense of proprioception in actual reality after taking off the VIP headsets. She mentioned, “*My only concern is if it [the VIP] makes the person dizzy, or if they have balance issues, depending on what you are looking at, it [the VIP] can make you feel dizzy.*” Staff and family members expected the VIPs to consider measures to address the challenges related to transitioning residents from the virtual immersive program to actual reality due to disturbances in care routines, being overwhelmed by the immersiveness, and having feelings of disorientation.

### Technology and usability barriers

Staff believed that technical barriers would reduce the applicability of the VIPs. Staff positioned themselves as an integral part of the VIP experience, even while not being immersed in it directly. The usability of the program was not framed as a resident-only issue, but as a shared process where staff competency directly shaped residents’ engagement. Staff considered their technical ability to navigate the equipment a determining factor of how smooth and enjoyable the experience would be for residents. Several staff members reflected on their own learning curve of their previous experience using VIP headsets, noting that it was not always intuitive to operate the system or to set it up efficiently for resident use. A staff member, Victoria stated,

It took me a while to get in there [the system] and figure it out, and then how am I gonna do this with people? Like, how am I gonna be able to go in [to the system] and find something and then put it [the video] on them [the VIP headsets].

Some staff voiced concerns that even with their support, VIPs would remain inaccessible to some residents, such as individuals with advanced dementia. As one staff participant, Palak described,

I'd really like them [residents] to be included and immersed in virtual reality, but when we explain it [VIP] to them, for people with more severe dementia, they may have a hard time understanding. Even with our help, there are only selective residents who can participate in this [immersive program].

Staff further highlighted their doubts on engaging residents who might not fully understand what they were seeing or the VIP technology. For example, residents might not realize that the content being viewed was not physically present or accessible. Even if residents understand, they may be unable to communicate that they understand and want to participate, leading staff members to perceive themselves as needed mediators when using VIPs with residents and compromising the autonomy of residents. A staff member, Evan, explained, *“It’s very hard to teach somebody [the residents] and say to them, “We’ll go over here [a spot in the VIP] and go there [another spot in the VIP],” but you are not in [the VIP experience].”* With VIPs offered through headsets particularly, the need to guide residents through a sensory world that staff could not simultaneously inhabit intensified staff concerns about using VIP with residents living with dementia. The gap between what staff could see and what residents were experiencing created a significant barrier to participation.

## Discussion

Our study uniquely contributes to the discourse on technology, co-design, and dementia care by investigating the pre-implementation expectations of all key end-user groups—residents, family members, and staff—for a virtual immersive program in long-term care. Guided by the empathy and define stages of the design thinking framework, our findings provide a critical foundational layer that is often overlooked in technology development, where studies typically evaluate outcomes after a program is already designed. We move beyond the established principle of “personalization” to reveal the nuanced tensions and specific desiderata that must be negotiated in a co-design process. Based on our findings, we propose the IMAGINE recommendations to help translate lessons learned into actionable guidance for creating VIPs that are ethical, inclusive, and person-centred. This will further be explored in the subsequent section outlining practical tips.

Our findings strongly corroborate the existing literature on the necessity of person-centred content to promote engagement and connection to identity (Liberman-Pincu and Oron-Gilad, 2025; Hodge et al., 2018; Matsangidou et al., 2023). Without personalizing VIPs, residents would be unable to feel connected to their identity, culture, nor actively engage with recalling memories (Ppali et al., 2025). While showcasing such content can bring positive feelings and improve quality of life as suggested by Graf et al. (2020) and Shin et al. (2023), there is an associated emotional risk of triggering negative emotions in residents (Meek et al., 2025). By seeing spaces and activities that residents previously enjoyed but are no longer able to participate in with the same capacity, residents may be reminded of their lack of agency in LTC as well as existing physical and cognitive limitations (Moyle et al., 2025; Fiocco et al., 2021). The lack of perceived autonomy, especially with residents with more advanced dementia, highlights the significance of engaging residents in decision-making more frequently by asking what a resident would like to see, as opposed to imposing assumptions on them. This echoes with an article by Hodge et al. (2018) that discussed that people with dementia should be active participants and the main driver of their experience. Additionally, while personalizing VIPs is highly beneficial for residents, individualizing content to niche preferences can undermine the feasibility of providing personally tailored content long-term (Goodall et al., 2021). In consideration of resource limitations in LTC, forming international partnerships with different organizations and volunteers, as well as effectively engaging family members in facilitating the production of individualized content can promote the practicality and sustainability of personally tailored content. Future studies can therefore explore engaging international partners and families in filming content for VIPs and how it affects output of individualized content.

Similar to other studies, our study noted that VIPs should show activities that residents enjoy (Rose et al., 2018; Appel et al., 2021; Woods et al., 2018; Manera et al., 2015; Matsangidou et al., 2025). Our participants further suggested the potential benefits to enhance residents’ engagement by featuring familiar faces in the VIP. However, specific ethical considerations may arise. For example, because of the possibility of triggering negative emotions, staff may need to discuss the potential tension and decide whether to show the video in the care home if the resident being featured passes away. There may also be privacy concerns as some individuals may not want to be shown on a VIP for the diverse populations in LTC indefinitely, particularly when the program is repetitively reviewed by end-users in an iterative process during the testing stage. Following on the importance of personally tailored content for each person, future research can explore the experiences of having videos with familiar faces and identify the potential facilitators and challenges of adopting these programs in LTC homes. Studies entering the prototyping and testing stages should additionally consider thorough consenting processes with individuals being featured on the VIP indefinitely at LTC homes. Moreover, while staff highlighted the opportunity to incorporate VIPs into care routines, such as bathing, it would be worthwhile to explore whether the integration creates comfort or further confusion for residents navigating the virtual world and the physical sensations from care staff. For example, bathing with VIPs involves combining multiple sensory stimulations, which may support distraction but may also lead to sensory mismatch and cognitive overload.

Similar to how our study participants anticipated challenges in utilizing and engaging residents in VIPs due to transitions between virtual and actual reality and expected technical barriers, some studies have likewise mentioned that disorientation in place and time were barriers in usage of the virtual programs (Matsangidou et al., 2025; Tabbaa et al., 2020; Matsangidou et al., 2020). However, the potential of VIPs to be disruptive in daily routines or anticipation of residents’ technology dependence was not mentioned in the literature to our knowledge. In the ideate phase of the co-design process, participants can co-plan strategies that may address these anticipated challenges which align with the care homes’ routines and philosophy of care.

A communal format of VIPs encourages dialogue between residents, staff, and family members while having the capacity to connect individuals from different backgrounds regardless of whether the program was tailored to their interests or culture. As Wong et al. (2025) mentioned, positive meaningful relationships can be strengthened when VIP experiences are shared between residents, staff, family members, and volunteers. Two other studies have reported similar social benefits of multiuser-experience virtual programs having the capacity to build a shared understanding and appreciation of each other’s experiences (Meek et al., 2025; Flynn et al., 2022). Similar to a previous study by Wong et al. (2024), staff members in LTC suggested to group residents and facilitators to watch a program together based on cultural similarities to streamline communication and cultural connection. While it is important to account for the cultural relevancy of residents, it is essential for facilitators to critically reflect on inclusivity and not to unintentionally exclude residents of diverse cultural backgrounds who may also be interested in viewing a program designed for a particular cultural group. When entering the prototyping and testing stages of the co-design process, it will be valuable to explore how residents from diverse cultural backgrounds respond to content tailored for a particular cultural group. Likewise, displaying content personalized to specific residents in a communal setting may compromise the extent of personalization to other residents participating in the VIP. In order to respect the dynamics between personalization and communal benefits, it may be beneficial to offer VIPs in a hybrid model where residents who prefer personalized content can view VIPs through headsets while in a space for VIP projections to optimize opportunities for social interaction. Future studies may consider intermixing both headsets and immersive projection use in a combined space and explore how this impacts the experience of end-users.

Besides cultural relevancy, our results show that staff see themselves as gatekeepers on which residents are “able” to join the VIP. Their decisions are based on their evaluations of residents’ capability to understand and engage. This may potentially exclude certain residents, such as those with later stages of dementia, from experiencing the program. This potential exclusion forms a barrier for residents to participate in the programs, which contrasts with previous studies highlighting staff as key facilitators in uptake of a VIP (Hung et al., 2023a; Jawharieh et al., 2025; Moyle et al., 2018). Wong et al. (2024) suggested that staff may unintentionally exclude some residents if there are perceived concerns of residents potentially damaging expensive equipment, while our study suggests that residents are uninvited if cognitive levels are further declined. To ameliorate the lack of agency in participation for residents, LTC homes can consider implementing education or regular discussion sessions to reflect on unconscious biases and take collaborative action to promote resident autonomy and personhood. Staff turnover in LTC settings is prevalent in Canada and results in lower qualities of care and high organizational costs, with one cross-sectional study particularly finding that 58% of LTC staff in Canada contemplate over quitting (Virdo and Daly, 2025). With insufficient time and staff shortages to personally learn about residents, LTC homes can recruit personnel such as volunteers to support participation and allow residents the opportunity to decide if they want to participate. At the same time, staff assumptions can create challenges on what is determined as meaningful to residents who are particularly in later stages of dementia. In a study investigating the psychosocial impacts of a VIP on residents, inclusion of family members who are very familiar with the residents helped extend the inclusivity of VIP to individuals who have increased cognitive decline or have challenges expressing themselves (Wong et al., 2025). Family members also tend to feel excluded in the care of their relatives in LTC (Baumbusch and Phinney, 2014). Therefore, future research should engage the family members of individuals with capacity challenges in the co-design process to promote inclusivity. Bryant et al. (2024) similarly shared in their discussion that older adults with communication impairments have the capacity to understand VIP systems with the appropriate support and training. While it is important to be considerate of how residents may perceive an experience, selectively imposing biases may prevent residents from acquiring potential benefits of the program. Therefore, in addition to acknowledging different stages of dementia through an empathetic lens, cultivating a personal relationship built on trust with residents is important to understand residents’ capabilities and histories to ensure that the opportunity of a VIP experience is equitable and inclusive. Future studies using a design thinking framework can co-plan additional creative strategies to learn about residents’ needs such as through self-documentation or observation (Lorusso et al., 2021).

### Practical tips

With respect to expected positive outcomes and desires of a VIP, participants have reported their desire for VIPs to have personalized content and interactions, enable exploration outside LTC, and support for care and communal experiences. The anticipated concerns of a VIP include over-engagement and immersion, and challenges in technological literacy hindering access for residents and staff engagement. Reflecting on the expectations and concerns of end-users in LTC, our team suggests practical tips for care homes or future research studies continuing to the ideate, prototype, and testing stages in co-designing similar VIPs using the following recommendations.

**IMAGINE**:

- **I**nclude residents in shared decision-making and respect residents’ agency
- **M**otivate staff and family facilitators to be present and prepare for potential triggers
- **A**dapt delivery and content to ensure that it’s engaging, meaningful, and inclusive
- **G**ive residents opportunities to try and avoid imposing personal biases
- **I**ntrospect on personal assumptions regularly to ensure authentic inclusivity
- **N**avigate technology dependency and strategies for transitions between virtual and actual reality
- **E**mpathize with the personhood of each participant and appreciate their diversity in experience, background, and capacities

### Strengths and limitations

Our paper includes diverse voices from residents with dementia that are often socially marginalized, as well as perspectives of family members and staff which are typically not included in research studies geared towards programs for residents in LTC. We engaged patient and family partners who provided their lived experiences in our research process, including the data analysis phase. However, our data was collected from a small participant sample size from one LTC home in British Columbia, Canada, which may not be representative of the broader Canadian population, as well as individuals in rural and remote regions within or outside Canada. Valuable insights from other residents, family members, and staff may have been accordingly missed. There is also a lack of cultural representation among participants due to the residents’ demographics in the two LTC homes we worked with. We may have missed insights specific to other cultural groups.

## Conclusion

Virtual immersive programs in LTC settings are a potential tool in improving the wellbeing and quality of life in older adults with dementia. Based on the first two stages of the design thinking framework, *empathize and define*, our research team explored expectations regarding content and delivery, and anticipated positive outcomes and challenges of a virtual immersive program in LTC with residents, family members and staff to tailor the program to the needs and preferences of end-users. Our suggested recommendations, IMAGINE, can support future studies and LTC leadership teams in co-designing a tailored and inclusive virtual immersive program for residents with dementia in LTC homes.

## Data Availability

The raw data supporting the conclusions of this article will be made available by the authors, without undue reservation.
